# Characterisation of RNA guanine-7 methyltransferase (RNMT) using a small molecule approach

**DOI:** 10.1042/BCJ20240608

**Published:** 2025-02-17

**Authors:** Lesley-Anne Pearson, Alain-Pierre Petit, Cesar Mendoza Martinez, Fiona Bellany, De Lin, Sarah Niven, Rachel Swift, Thomas Eadsforth, Paul Fyfe, Marilyn Paul, Vincent Postis, Xiao Hu, Victoria H. Cowling, David W. Gray

**Affiliations:** 1 Drug Discovery Unit, School of Life Sciences, University of Dundee, Dundee, U.K.; 2 School of Cancer Sciences, University of Glasgow, Garscube Estate, Glasgow, U.K.

**Keywords:** breast cancer, methyl transferase, RNMT, Sinefungin

## Abstract

The maturation of the RNA cap involving guanosine N-7 methylation, catalyzsed by the HsRNMT (RNA guanine-7 methyltransferase (HsRNMT)-RAM (RNA guanine-N7 methyltransferase activating subunit (RAM) complex, is currently under investigation as a novel strategy to combat PIK3CA -mutant breast cancer. However, the development of effective drugs is hindered by a limited understanding of the enzyme’s mechanism and a lack of small molecule inhibitors. Following the elucidation of the HsRNMT-RAM molecular mechanism, we report the biophysical characterizsation of two small molecule hits. Biophysics, biochemistry and structural biology confirm that both compounds bind competitively with cap and bind effectively to HsRNMT-RAM in the presence of the co-product SAH, with a binding affinity (K_D_) of approximately 1 μM. This stabilisation of the enzyme-–product complex results in uncompetitive inhibition. Finally, we describe the properties of the cap pocket and provided suggestions for further development of the tool compounds.

## Introduction

**Figure 1:**
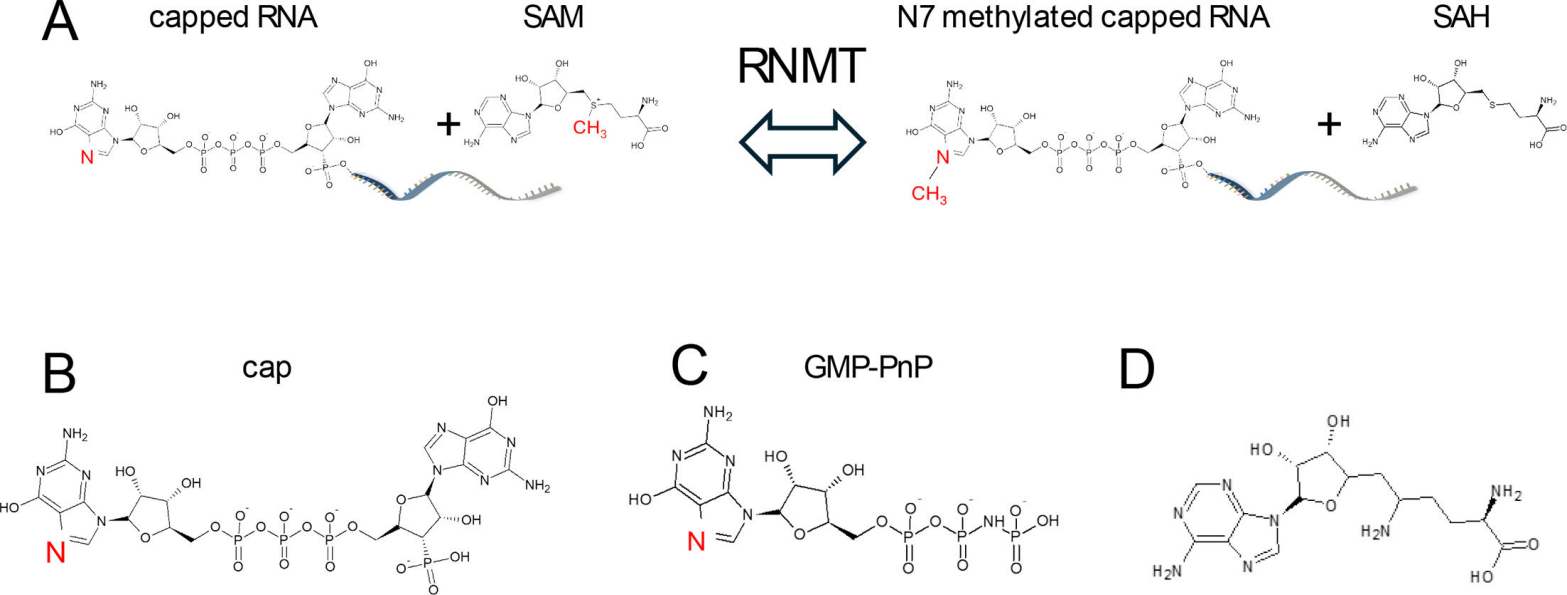
Diagram of enzymatic reaction and structures of key substrate and analogues. (**A**) Diagram of enzymatic reaction carried ofout by RNMT. The N7 nitrogen on the cap and the methyl group are highlighted in red. (**B**) The cap structure minus RNA as used in the present paperstudy. (**C**) GMP-PnP as used as a cap surrogate in the crystallography experiments and (**D**) Sinefungin, a non-selective methyltransferase inhibitor acting as a SAM mimetic. Abbreviations: GMP-PnP, guanosine 5’-[β,γ-imido]triphosphate; RNMT, RNA guanine-7 methyltransferase; SAM, S-adenosylmethionine.

RNA polymerase II transcripts are modified at the 5′ end by the co-transcriptional addition of the RNA cap [1,2]. In mammals, this structure is 7-methylguanosine linked by a 5′-to-5′ triphosphate to the first-transcribed nucleotide. The first- and second-transcribed nucleotides can be further methylated by other methyltransferases [3,4]. The RNA cap is formed during the early stages of transcription as the nascent RNA emerges from the polymerase complex [1,4]. RNA guanylyltransferase and 5′ phosphatase binds directly to the RNA pol II large subunit and catalyses the addition of the guanosine cap to the nascent transcript, denoted as G(5′)ppp(5′)X, where X is the first-transcribed nucleotide. RNA guanine-7 methyltransferase (RNMT) catalyses the transfer of a methyl group from S-adenosylmethionine (SAM) to the cap intermediate to form the cap m7G(5′)ppp(X) and the by-product S-adenosylhomocysteine (SAH) (Figure 1A).

RNMT was identified based on homology to the yeast and viral homologues and *in vitro* activity [5,6]. This basic cap structure (cap 0, m7G(5′)ppp(X)) protects RNA from degradation by nucleases during synthesis and recruits factors involved in RNA processing and translation initiation [1,2]. RNMT has an activating co-factor RNMT-activating miniprotein (RAM) clamped at the interface of the catalytic domain and the modular lobe 416–456 [7]. This stabilisation promotes SAM recruitment and induces a significant increase in the methyltransferase activity [8,9] through the formation of a series of interactions affecting the α-helix hinge, α-helix A and multiple active site residues [10]. RAM has also an RNA-binding domain that recruits RNA to RNMT [9,11]. RNMT-RAM is critical for gene expression [12,13]. In addition to its role of catalysing RNA cap methylation during transcription and later, RNMT-RAM has also non-catalytic roles in transcription and potentially other processes [11,14]. Deletion of RNMT results in reduced proliferation and increased apoptosis, and deregulated expression of RNMT promotes cell transformation [15–18].

Several cellular signalling pathways regulate RNMT by modulating the expression of RNMT itself or activating subunit RAM, by altering its activity through post-translational modifications or by controlling metabolism of SAH, the inhibitory by-product of methylation reactions [1,19–21]. The regulation of RNMT accompanies or drives major cellular transitions and directs enhanced expression of specific sets of genes. For example, RNMT-RAM is up-regulated during T-cell activation when rapid cell growth and proliferation requires increased mRNA synthesis for adaptive immune responses [12]. Of particular importance is the enhanced production of TOP-RNAs that encode ribosomal proteins and other proteins involved in ribosome production. Conversely, during embryonic stem cell differentiation, RNMT-RAM is down-regulated, which is required for the repression of pluripotency-associated genes and morphological features of differentiation [19].

Consequently, RNMT is a target of interest in cancers and immune disorders. These diseases exhibit deregulated transcription, which requires a cap, and RNMT inhibition has been demonstrated to reduce proliferation and increase apoptosis in many cell lineages [8,16,21]. Certain oncogenic mutations increase dependency on the cap. Breast cancer cell lines with oncogenic PIK3CA mutations have enhanced sensitivity to RNMT inhibition [15]. Myc-dependent transcription and proliferation is dependent on RNA cap formation [20,22,23]. Recent analyses have linked RNMT expression to cancer outcomes [24,25].

Here, we use biophysical, biochemical and structural biology approaches to build understanding of the mode of enzymatic action of RNMT. Further, by carrying out a diversity screening campaign, we describe two novel, small molecule inhibitors of RNMT that may be developable into useful tools for interrogating the role of RNMT in different cell contexts.

## Results

**Figure 2:**
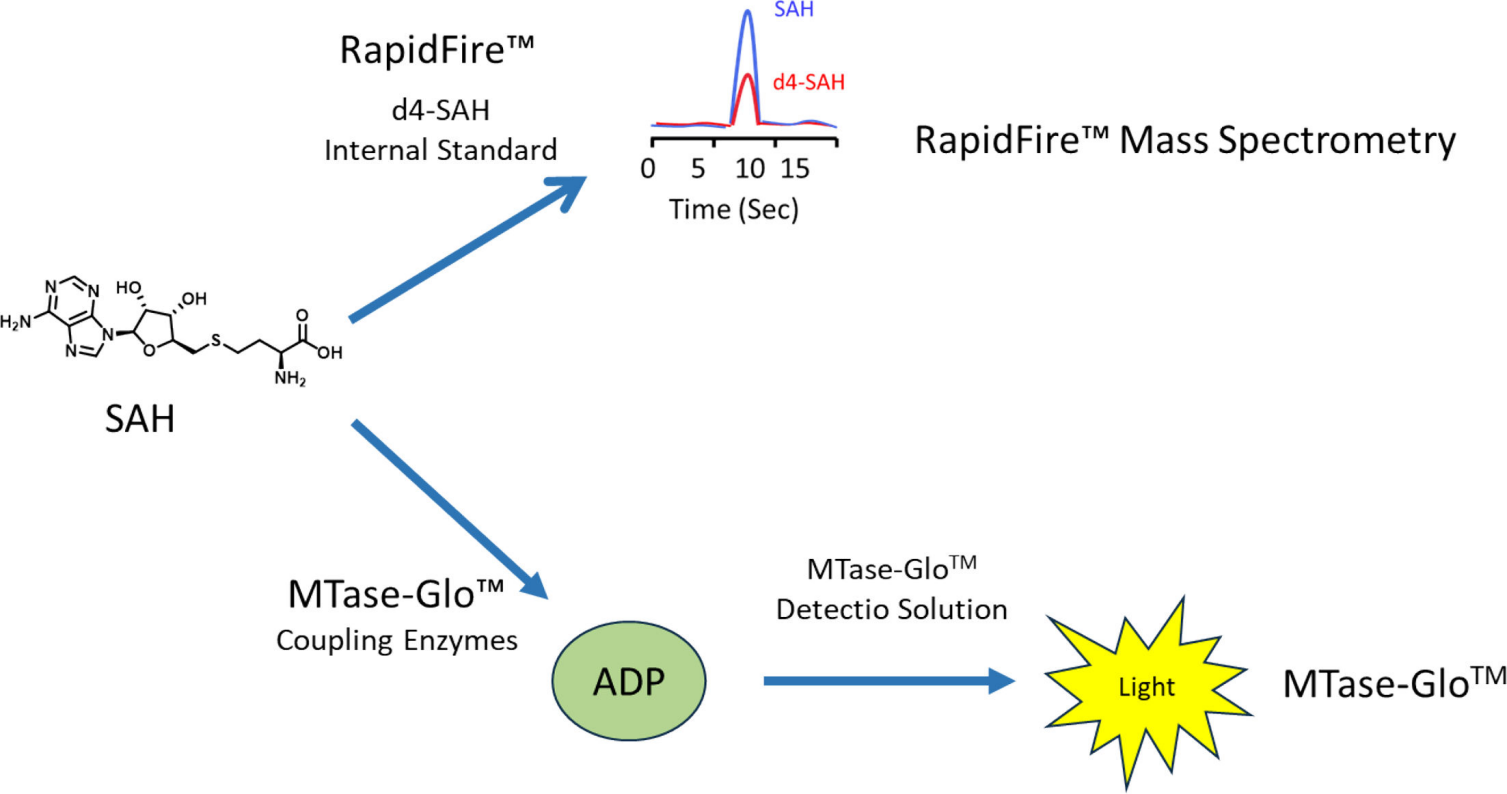
Diagram comparing the two assay formats developed. Both measure SAH. The MTase-Glo™ assay uses coupling enzymes to convert SAH to a light readout. The RapidFire™ mass spectrometry assay measures SAH levels directly. Abbreviation: SAH, S-adenosylhomocysteine.

In this manuscript, cap is GpppG (Figure 1B), except in crystallographic studies where a cap surrogate guanosine 5′-[β,γ-imido]triphosphate (GMP-PnP) (Figure 1C) was used. Sinefungin (Figure 1D) is used as a SAM mimetic [26]. This molecule has been shown to bind in the active site of a wide range of methyltransferases with substrates as diverse as DNA [27], RNA [28], glycopeptides [29] and catecholamine synthesis enzymes [30]. Methylated cap and SAH are the final products of the enzymatic process. Two RNMT biochemical assays were developed with different readouts (Figure 2).

The first utilises the MTase Glo reagent (Promega) to convert the product of the methyltransferase assay into a bioluminescent signal [31] through coupling enzymes. We found that this assay had some susceptibility to false positives, presumably through inhibition of the coupling enzymes rather than RNMT. In view of this, we developed a mass spectrometry readout based on the accumulation of the product, SAH, using the RapidFire system. Both assays share the same basic biochemical setup with enzyme and substrate concentrations, and time-courses being similar. However, the mass spectrometry assay is stopped with formic acid and the plates sealed. The RapidFire processing system allows the sample to be aspirated from the well and loaded on to a solid-phase extraction column where the buffer salts and proteins are removed by washing with a TFA solution. The SAH was eluted using an acetonitrile:water:TFA solution and directed to a triple quadrupole mass spectrometer (Agilent 6740). Full details of the SAH RapidFire assay protocol have been published using a different methyltransferase [32].

### Biophysical and biochemical characteristics of RNMT

**Figure 3:**
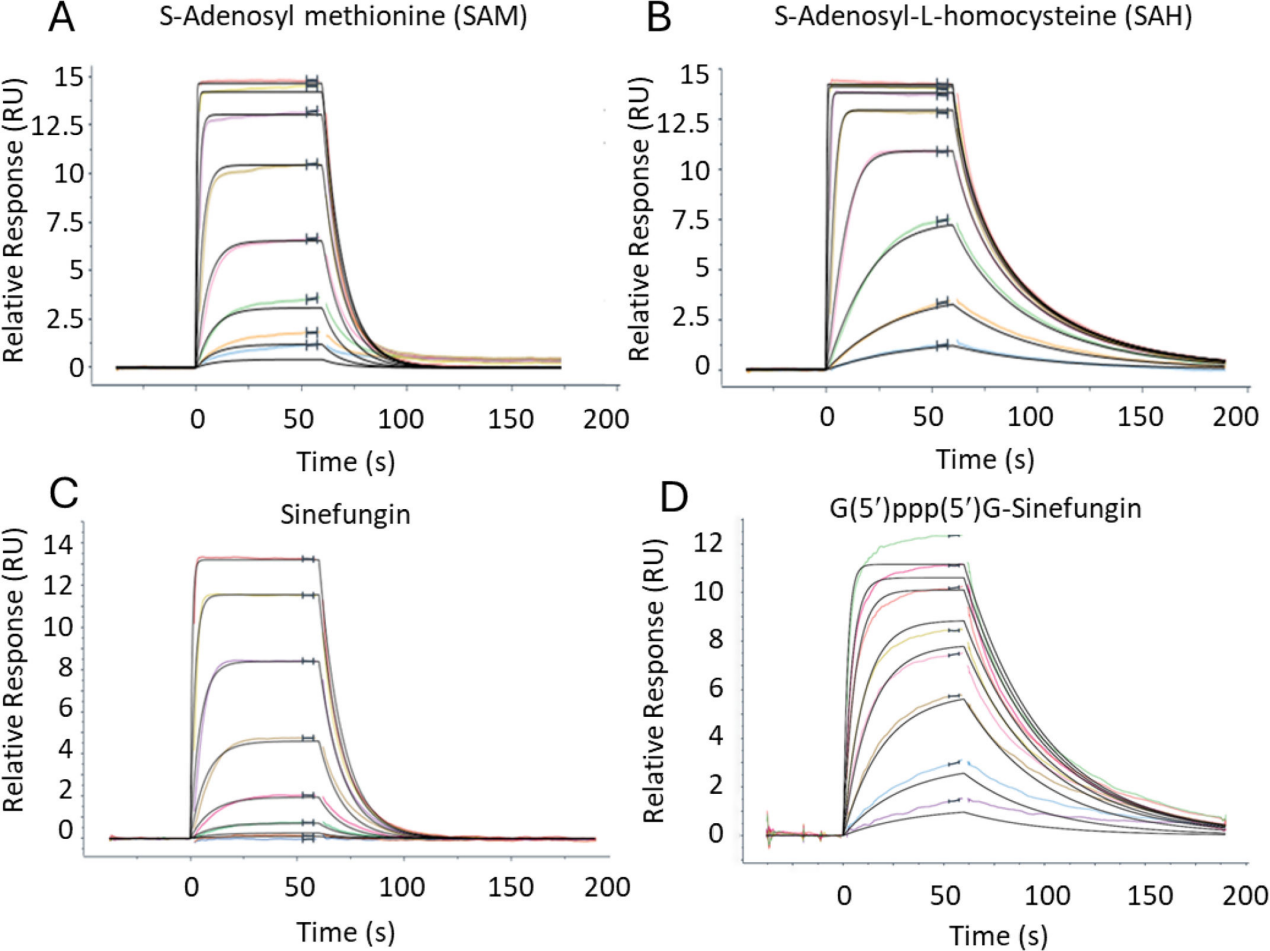
Sensograms derived from SPR-binding experiments. All experiments were conducted with eight concentrations. SAM: - top concentration 5 mM, 1 in 3 dilutions (**A**), SAH: – top concentration 5 mM, 1 in 3 dilutions (**B**), Sinefungin: – top concentration 0.5 mM, 1 in 2 dilutions (**C**), or G(5′)ppp(5′)G-Sinefungin: – top concentration 0.37 mM, 1 in 3 dilutions were flowed over immobilizsed RNMT-RAM complexes. Data isare representative of 2two experiments. Data wasere processed using Langmuir 1:1 model and isare summarizsed in Table 1. Abbreviations: RAM, RNMT-activating miniprotein; RNMT, RNA guanine-7 methyltransferase; SAH, S-adenosylhomocysteine; SAM, S-adenosylmethionine; SPR, surface plasmon resonance.

To elucidate the cap-binding mode, full-length *Hs*RNMT in complex with activating subunit RAM (Avi-HsRNMT-RAM) was investigated by surface plasmon resonance (SPR). We first measured the kinetic and equilibrium binding constants (K_D_) for SAM (Figure 3A), SAH (Figure 3B), and Sinefungin (Figure 3C). The kinetics of binding for these compounds appear very similar with a rapid onset of binding reaching a steady state within a few seconds. The loss of binding on washing with buffer was very similar.

**Table 1:** Summary of association (*ka*), dissociation (*kd*) and affinity (K_D_) estimates from the SPR assays.

Titrated ligand	SAM	SAH	Sinefungin	Cap	Cap	Cap
Ligand titrated in presence of	-	-	-	-	SAH	Sinefungin
*ka* (×10^6^M^-1^s^-1^)	1.2	3.8	1.2	No binding	No binding	1.2
*kd* (×10^-2^s^-1^)	10	7.2	9.2			2.5
Rmax (RU)	14.9	14.3	14.2			11.8
K_D_ (nM) (kineti*c*)	79	19	77			20
K_D_ (nM) (steady state)	89	18	73			35

Rmax indicates the maximum binding capacity of the titrated fragment. Data were processed using a Langmuir 1:1 model.

SAH, S-adenosylhomocysteine. SAM, S-adenosylmethionine. SPR, surface plasmon resonance.

No interaction of the cap with *Avi-HsRNMT-RAM* was detected in the absence of co-substrate or in the presence of SAH. The interaction of the cap with *Avi-HsRNMT-RAM* was only detected after addition of a saturating concentration (1 µM) of Sinefungin to the assay buffer (Figure 3D). Kinetic and steady-state calculations of affinity constants were consistent. These biophysical results are summarised in Table 1.

Altogether, these results suggest that *Hs*RNMT-RAM has an ordered binding mechanism with SAM binding first, followed by the cap. Furthermore, biophysical characterisations also suggest that the interaction of the cap with *Hs*RNMT-RAM was driven by the 5-amino group on Sinefungin (or the SAM methyl group in a physiological condition) as SAH does not carry a comparable group at position 5 and no cap binding was detected. We hypothesise that in the absence of a 5-amino/methyl group, the cap guanine moiety is rotated by 180° in a clockwise direction, a consequence of the intra-molecular attractive force between the exocyclic -NH_2_ of the guanine moiety and the phosphates. This conformation would be detrimental to the cap binding since most of the interactions with *Hs*RNMT-RAM would be lost.

**Figure 4:**
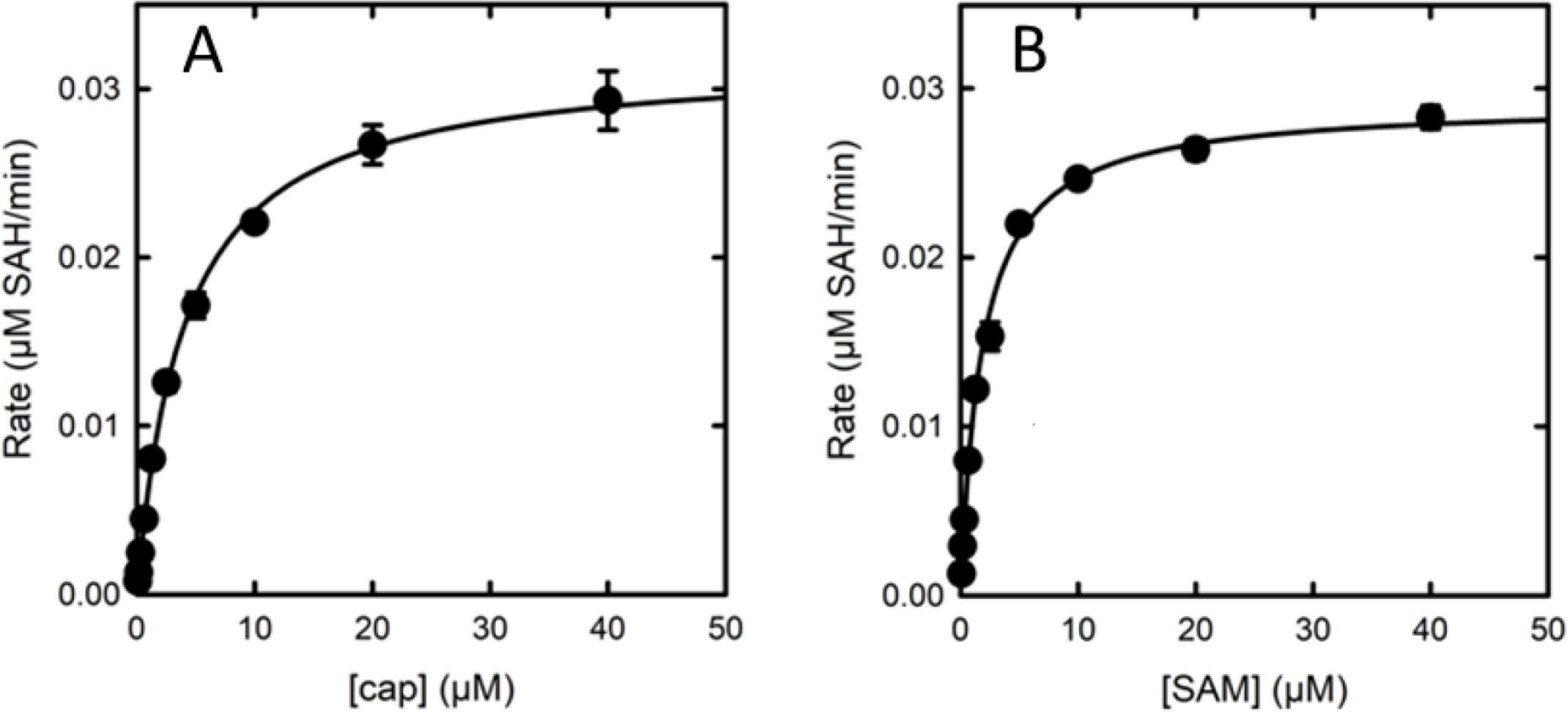
Michaelis–Menten plots for *Hs*RNMT-RAM substrates. cap (40–0.078 µM) (**A**) and SAM (40–0.078 µM) (**B**). Plots shown are representative examples from three experiments. Error bars are ± standard deviation from four technical replicates. Abbreviations: RAM, RNMT-activating miniprotein; RNMT, RNA guanine-7 methyltransferase; SAM, S-adenosylmethionine.

Michaelis–Menten parameters were determined for *Hs*RNMT-RAM using the RapidFire Mass Spec assay for cap (*K*_M_^cap^ = 3.2 µM [2.0–5.3 µM]), SAM (*K*_M_^SAM^ = 1.6 µM [1.3–2.1 µM]), and a mean *V*max 0.04 µM (0.04–0.05) SAH/min for both substrates (Figure 4). These data provide assay conditions suitable for characterisation of inhibitors.

### Screening campaign

**Figure 5:**
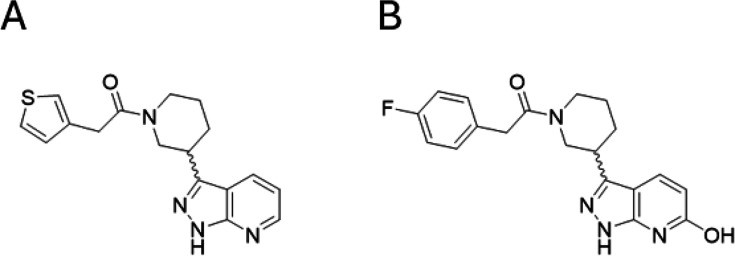
Chemical structures of (**A**) DDD1060606 and (**B**) DDD1870799. DDD1060606 and DDD1870799 showed >80% inhibition of *Hs*RNMT-RAM activity with determined pIC_50_s (negative logarithm of half-maximal inhibitory concentration [molar]) of 5.5 ± 0.2 (IC_50_ 3 mM; Figure 6D and 5.0 ± 0.2 (IC_50_ 10 mM; Figure 6G), respectively. Abbreviations: RAM, RNMT-activating miniprotein; RNMT, RNA guanine-7 methyltransferase.

Following the establishment of appropriate ‘balanced’ assay [33], a screening campaign was carried out on 48,806 diverse drug-like compounds [34] from our internal compound library at a single concentration (10 µM) using the MTAse Glo assay to measure enzymatic activity. Analysis of the single-point screen revealed a normal distribution ( Supplementary Figure S1) with a mean inhibition of 4.6% and standard deviation of 15.4%. Using a cut-off of 50.8% (mean + 3 × standard deviation; 99.73% confidence assuming a normal hit distribution), the *Hs*RNMT-RAM assay resulted in a hit rate of 1.6%. The screen was robust with a mean Z′ [35] of 0.8 (±0.1) and mean signal:background of 7.7 (±3.6). Two compounds with similar structures (Figure 5) were profiled in the orthogonal RapidFire assay.

**Figure 6:**
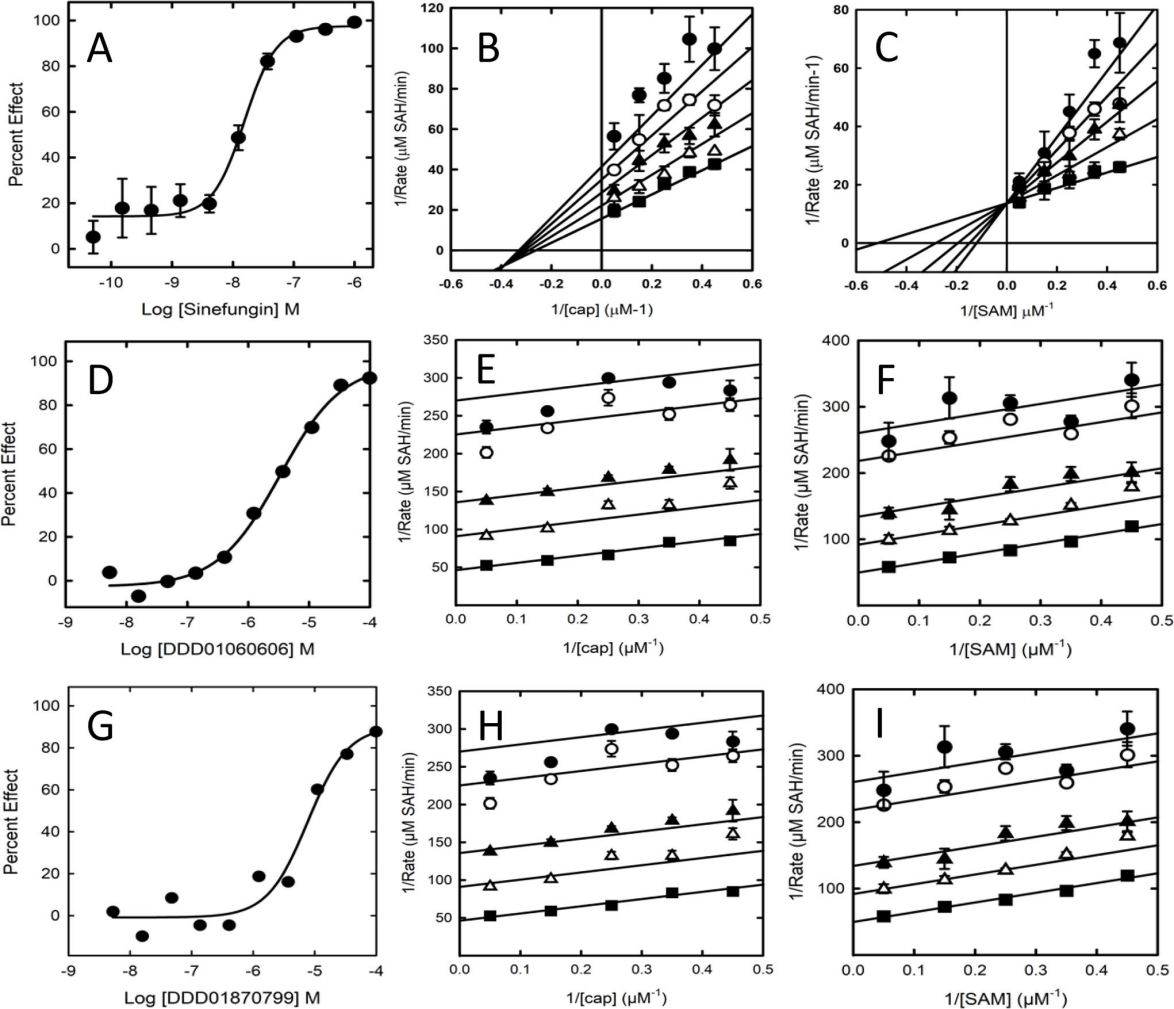
Characterisation of inhibition by Sinefungin, DDD1060606 and DDD1870799. (**A**) is tThe inhibition curve for Sinefungin (10 point 1:3 dilutions starting at 1 μM, duplicates on assay plate). (**B**) The Sinefungin ((0 (■), 40 (△), 80 (▲), 120 (◯) and 160 nM (●)) Lineweaver–-Burk plot against cap. and (**C**) The Sinefungin ((0 (■), 8 (△), 16 (▲), 24 (◯) and 32 μM (●)) Lineweaver-–Burk plot against SAM. (**D–F**) The inhibition curve (10 point 1:3 dilutions starting at 100 μM, singlicates on assay plate) and Lineweaver-–Burk plots against cap and SAM, respectively, for DDD1060606 (0 (■), 1.25 (△), 2.5 (▲), 5 (◯) and 6.25 μM (●)). (**G–I**) The inhibition curve (10 point 1:3 dilutions starting at 100 μM, singlicates on assay plate) and Lineweaver-–Burk plots against cap and SAM, respectively, for DDD1870799 (0 (■), 1.25 (△), 2.5 (▲), 5 (◯) and 6.25 μM (●)). Inhibition curves and Lineweaver-–Burk plots are representative of 3three experiments. Error bars in the Lineweaver-–Burk plots are standard deviation from three technical replicates. Abbreviation: SAM, S-adenosylmethionine.

### Mode of inhibition

Mode of inhibition experiments were performed for Sinefungin, DDD1060606 and DDD1870799 using the RapidFire assay. While Sinefungin showed competitive inhibition with SAM (Figure 6C) and non-competitive inhibition with cap (Figure 6B) as was anticipated, both small molecule inhibitors demonstrated uncompetitive inhibition profiles against both cap and SAM (Figure 6E, F, H, I). The data from these experiments are summarised in Table 2.

**Table 2:** Summary of mode of inhibition and key inhibition constants for Sinefungin, DDD1060606 and DDD1870799.

Compound	K_i_ cap	K_i_ SAM
Sinefungin	Non-competitive 104.2 nM (92.2–117.8 nM)	Competitive 6.8 nM (3.1–14.9 nM)
DDD1060606	Uncompetitive 1.6 µM (0.4–5.7 µM)	Uncompetitive 1.6 µM (0.4–5.7 µM)
DDD1870799	Uncompetitive 3.3 µM (2.4–4.6 µM)	Uncompetitive 3.4 µM (2.8–4.2 µM)

Data are presented as K_i_ (95% CI).

### Compound binding analysis

**Figure 7:**
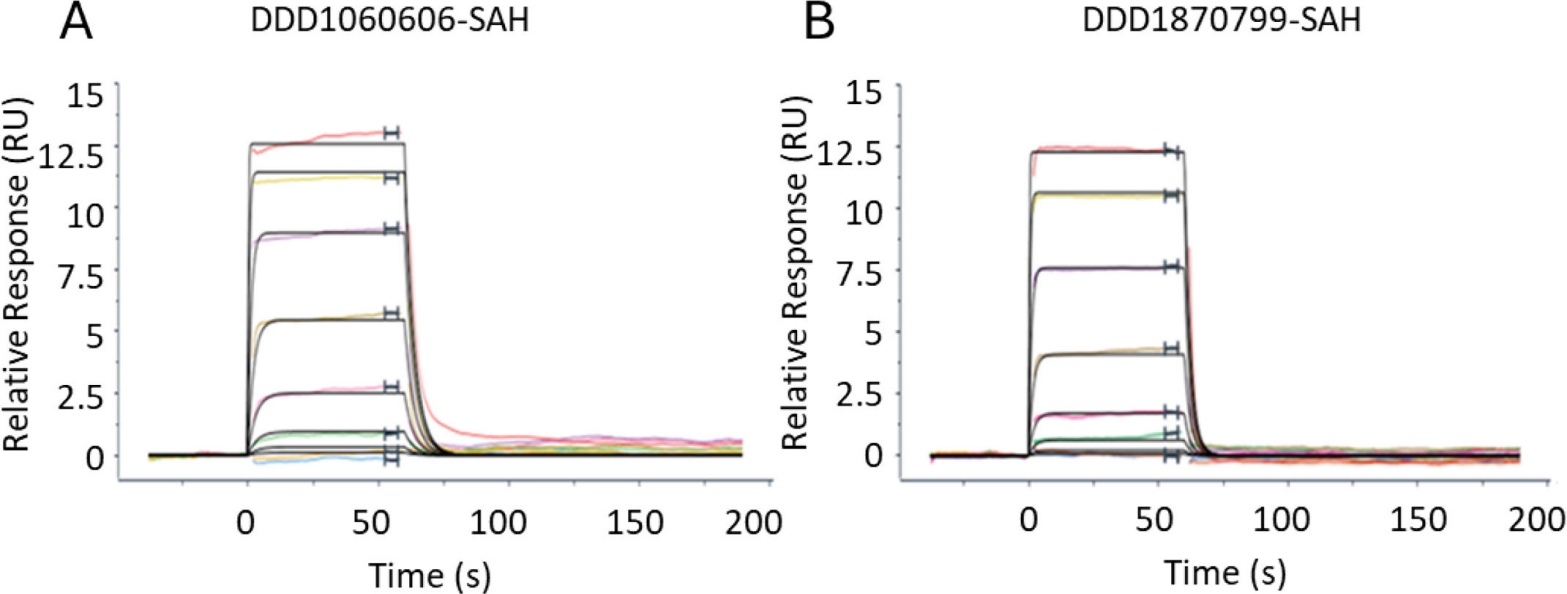
Sensograms derived from SPR binding experiments. All experiments were conducted with an eight concentration in a 1:3 serial dilution. DDD1060606: – top concentration 30 mM (**A**) or DDD1870799: – top concentration 30 mM (**B**) were flowed over immobilizsed RNMT-RAM complexes in the presence of SAH (5 mM). Data isare representative of 2two experiments. Data wasere processed using Langmuir 1:1 model and are summarizsed in Table 3. Abbreviations: RAM, RNMT-activating miniprotein; RNMT, RNA guanine-7 methyltransferase; SAH, S-adenosylhomocysteine; SPR, surface plasmon resonance.

We used SPR to investigate the co-factor requirements to permit the binding of the inhibitors to *Hs*RNMT-RAM. No binding was observed in the absence of co-ligand or in the presence of SAM, whereas high-affinity binding (K_D_ = 1.6 µM and 2.5 µM (Figure 7) was measured for DDD1060606 and DDD1870799, respectively, when the buffer was supplemented with SAH. This suggests that the ligands bind preferentially after the reaction has completed, and SAM has been metabolised into SAH. The data from these experiments are summarised in Table 3.

**Table 3:** Summary of association (*ka*), dissociation (*kd*) and affinity (K_D_) estimates from the SPR assays.

Titrated ligand	DDD1060606			DDD1870799		
Ligand titrated in presence of	-	SAM	SAH	-	SAM	SAH
*ka* (x10^6^M^-1^s^-1^)	No binding	No binding	17	No binding	No binding	18
*kd* (x10^-2^s^-1^)			27			45
Rmax (RU)			13.2			13.3
K_D_ (μM) (kinetic)			1.6			2.5
K_D_ (μM) (steady state)			1.4			2.3

Rmax indicates the maximum binding capacity of the titrated fragment. Data were processed using a Langmuir 1:1 model.

SAH, S-adenosylhomocysteine. SAM, S-adenosylmethionine. SPR, surface plasmon resonance.

### Crystallography

Previous work [7] demonstrated that the lobe 416–455 becomes highly agitated in the absence of RAM. While we were able to crystallise RNMT catalytic domain in the presence of RAM, the crystallisation process was insufficiently robust for drug discovery efforts. To improve the robustness of the crystallisation process, the lobe 416–455 of *Hs*RNMT catalytic domain was replaced by its homologous sequence region from the *Encephalitozoon cuniculi* (GLGC) guanine-7 cap methyltransferase (Q8SR66) identified from a pairwise sequence alignment. The sequences used are in Supplementary Figure S2. The two chains of the asymmetric unit for all structures are almost identical and can be well superposed (RMSD < 0.5 Å) to the structure of reference (5e8j) solved in complex with RAM [7] ( Supplementary Figure S3). This validates our hypothesis that the effect of this lobe substitution would be negligible on the RNMT-RAM structure.

## Cap binding analysis

**Figure 8:**
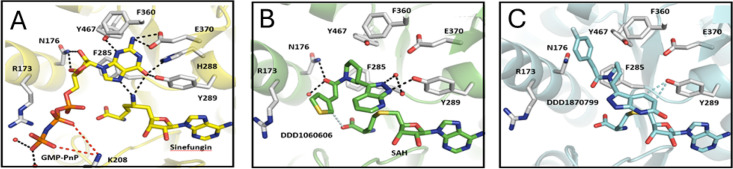
Structural analysis of the *Hs*RNMT cap binding site. (**A**) GMP-PnP in complex with Sinefungin, (**B**) DDD1060606 in complex with SAH and (**C**) DDD1870799 in complex with SAH. Residues within 4 Å are drawn in sticks. Dash lines represent hydrogen bonds (black), salt bridges (red) and aromatic H-bonds (cyan). Abbreviations: GMP-PnP, guanosine 5′-[β,γ-imido]triphosphate; RNMT, RNA guanine-7 methyltransferase; SAH, S-adenosylhomocysteine.

The understanding of the cap binding mechanism is currently limited by the absence of a published *Hs*RNMT-cap structure and the lack of knowledge of the key residues involved in cap binding. This deficit hinders the development of potent and/or selective drugs targeting the cap pocket. Since attempts to crystallise RNMT with the G(5′)ppp(5′)G (cap) had been unsuccessful, we co-crystallised RNMT with the cap surrogate, GMP-PnP. GMP-PnP was confirmed as a cap competitive inhibitor with a *Ki* of 2.3 µM (0.9–5.5 µM) (Supplementary Figure S5). Crystallisation of RNMT with GMP-PnP provides the first structural characterisation of a Class I methyltransferase in a complex with a cap analogue and Sinefungin. The Sinefungin molecule is well resolved in the electron density map (Supplementary Figure S6) for both chains of the asymmetric unit and shares a common network of interaction to that observed for SAH binding to *Hs*RNMT (Supplementary Figure S4). GMP-PnP was successfully built in just one of the chains in the asymmetric unit and is located between the α-helix A and the β-strands 8/9. When co-crystallised with HsRNMT and Sinefungin (Figure 8A), the GMP-PnP guanine moiety is stabilised within the cap pocket by hydrogen bonds to residues H288, Y289, E370, Y467 and the Sinefungin 5-amino group, as well as through a Pi-stacking interaction with F285.

The GMP-PnP ribose makes two hydrogen bonds with N176, whereas the beta-gamma pyrophosphate group interacts with the polypeptide chain via two salt bridges to K208. Finally, the distance measured between the Sinefungin 5-amino group and the guanine N7 (2.4 Å) alongside the angle formed by C5/5-NH2/N7 (140°) confirms that GMP-PnP is observed in an ideal orientation to permit the methyl transfer, similar to the predicted model published [10].

### SAM/SAH-binding site

*Hs*RNMT–Sinefungin–GMP-PnP, *Hs*RNMT–SAH–DDD1060606 and *Hs*RNMT–SAH–DDD1870799 co-complexes were solved, respectively, at 2.5, 2.0 and 2.4 Å in space group *P*2_1_ ( Supplementary Table S1).

In all the three structures, SAH or Sinefungin is observed in a hydrophobic pocket made by the α-helices 3 and 4 and the loops connecting the β-strand 1 to the α-helix B and the β-strands 3 and 4. SAH and Sinefungin are stabilised in the active site through an identical network of hydrogen bonds, involving residues K180, G205, D227, D261, S262 and Q284, as well as 2 salt bridges with K180 ( Supplementary Figure S3). These results support the hypothesis that Sinefungin binds in the SAM-binding site in the presence or absence of the cap.

The two inhibitors (DDD1060606 and DDD1870799) identified in the screening campaign were successfully co-crystallised with *Hs*RNMT in the presence of SAH. DDD1060606 (Figure 8B) is observed at the cap pocket and displays a mainly hydrophobic binding mode. Using the *Hs*RNMT–Sinefungin–GMP–PnP as a structure of reference, the pyridine is rotated 180° and sits on top of the SAH homocysteine moiety, making direct and water-mediated hydrogen bonds with Y289 and H288 side chains, as well as F285 main chain.

The piperidine is located nearby the GMP-PnP ribose and is mainly stabilised by a hydrogen bond with N176. Finally, the thiophene near to the GMP-PnP α-phosphoryl group is largely solvent exposed and does not interact with the peptide chain.

DDD1870799 (Figure 8C) is also observed in the cap pocket of RNMT. In the presented structure, the pyrazolo-pyridine interacts with Y289 via two aromatic H-bonds, while the fluoro-benzene is buried in a hydrophobic pocket made by the residues including the main chain R173, as well as the side chains N176 and Y467.

### Active-site characterisation

**Figure 9:**
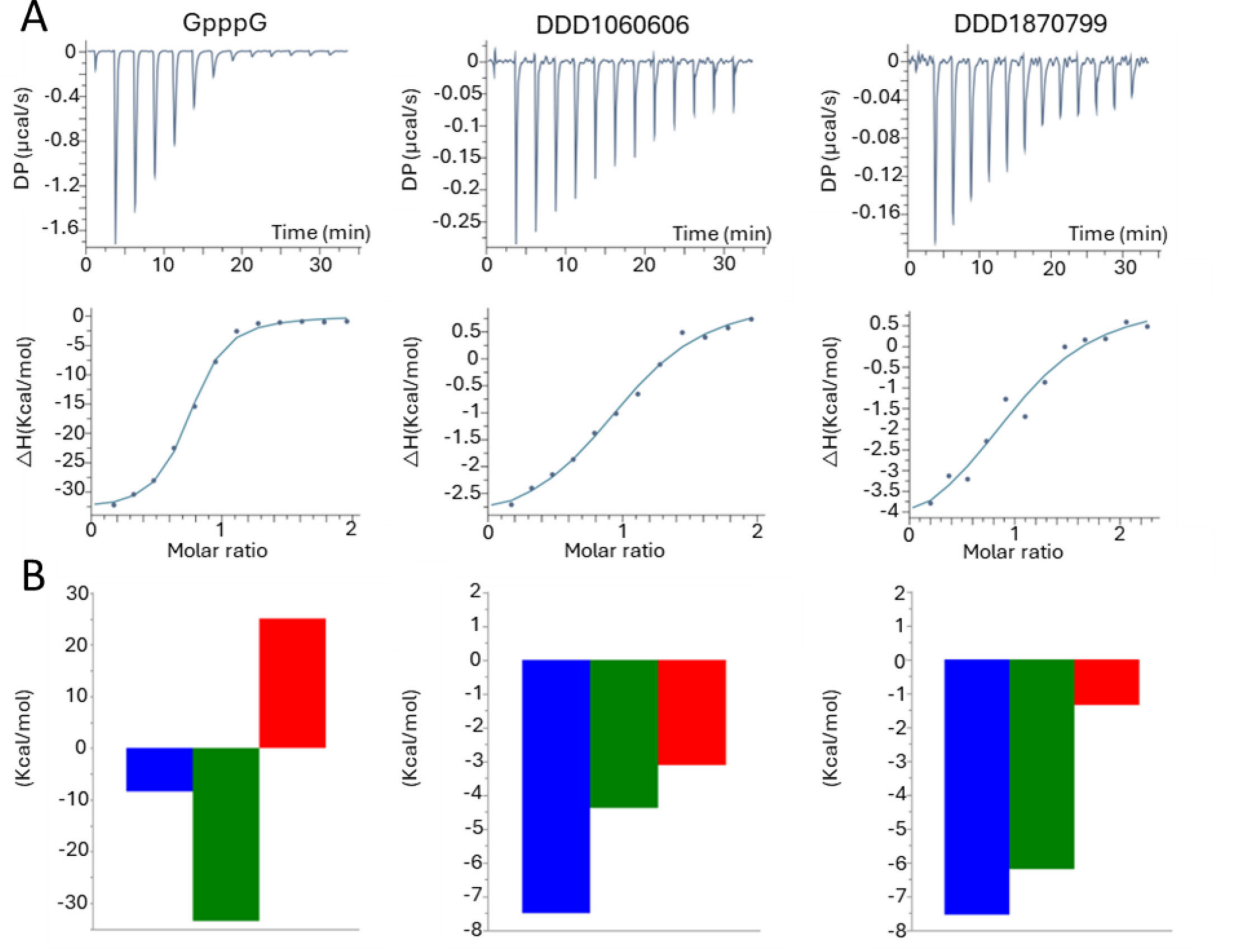
Cap, DDD1060606 and DDD1870799 titrations into RNMT-RAM. (**A**) Raw data and integration of data, corrected for the heat of dilution. The line represents the least-squares fit to the single-site binding model by the ORIGIN program. (**B**) Thermodynamic signatures (ΔG° in blue, ΔH° in green, − TΔS° in red, all data in Kkcal/mol). Abbreviations: RAM, RNMT-activating miniprotein; RNMT, RNA guanine-7 methyltransferase.

For both DDD1060606 and DDD1870799, we determined the enthalpic and entropic components of the free energy of binding to *Hs*RNMT-RAM to guide both compound selection and the optimisation process. Note that all of the isothermal titration calorimetry (ITC) experiments were performed with protein pretreated with SAH or Sinefungin. We used cap as the reference ligand as it is widely accepted that ligands with more negative enthalpies of binding provide better starting points for lead optimisation [36,37]. The interaction of cap with *Hs*RNMT-RAM is enthalpically driven with an ΔH of −33.5 kcal/mol (Figure 9A) and net free energy change, ΔG of −8.4 kcal/mol (Figure 9B). Furthermore, we determined the binding affinities for DDD1060606 and DDD1870799 to *Hs*RNMT-RAM.

The resulting affinities (both approximately 3 μM) are comparable to the K_D_ measured by SPR in the previous experiments. A favourable entropic contribution Δ(TΔS) of −1.8 kcal/mol was observed for DDD1060606, and the total Gibbs free energies were similar for both ligands (ΔG = −7.5 kcal/mol). However, a significant gain in net enthalpic change ΔΔH of −1.9 kcal/mol was measured for DDD1870799 in comparison with DDD1060606, suggesting that the interaction of DDD1870799 with *Hs*RNMT-RAM is more enthalpy driven and might result in more selective leads with fewer absorption, distribution, metabolism, excretion and toxicity issues than DDD1060606 [38,39] (Figure 9). The data from this analysis are summarised in Table 4.

**Table 4:** Summary of mode of inhibition and key inhibition constants for Sinefungin, DDD1060606 and DDD1870799.

Ligand	ΔG (kcal/mol)	ΔH (kcal/mol)	−TΔS (kcal/mol)	N	K_D_ (μM)
Cap	−8.4	−33.5	25.1	0.7	0.7
DDD1060606	−7.5	−4.3	−3.1	1.0	3.2
DDD1870799	−7.5	−6.2	−1.3	1.0	2.9

Isothermal titration calorimetry titrations were performed in 25 mM HEPES pH7.5, 150 mM NaCl, 1 mM TCEP supplemented with 10 mM Sinefungin or SAH at 298°K.

We then calculated the molecular interaction fields (MIFs) by FLAP [40] to identify the attributes that lead to the biological activity of the cap analogue and the 2 new inhibitors.

**Figure 10:**
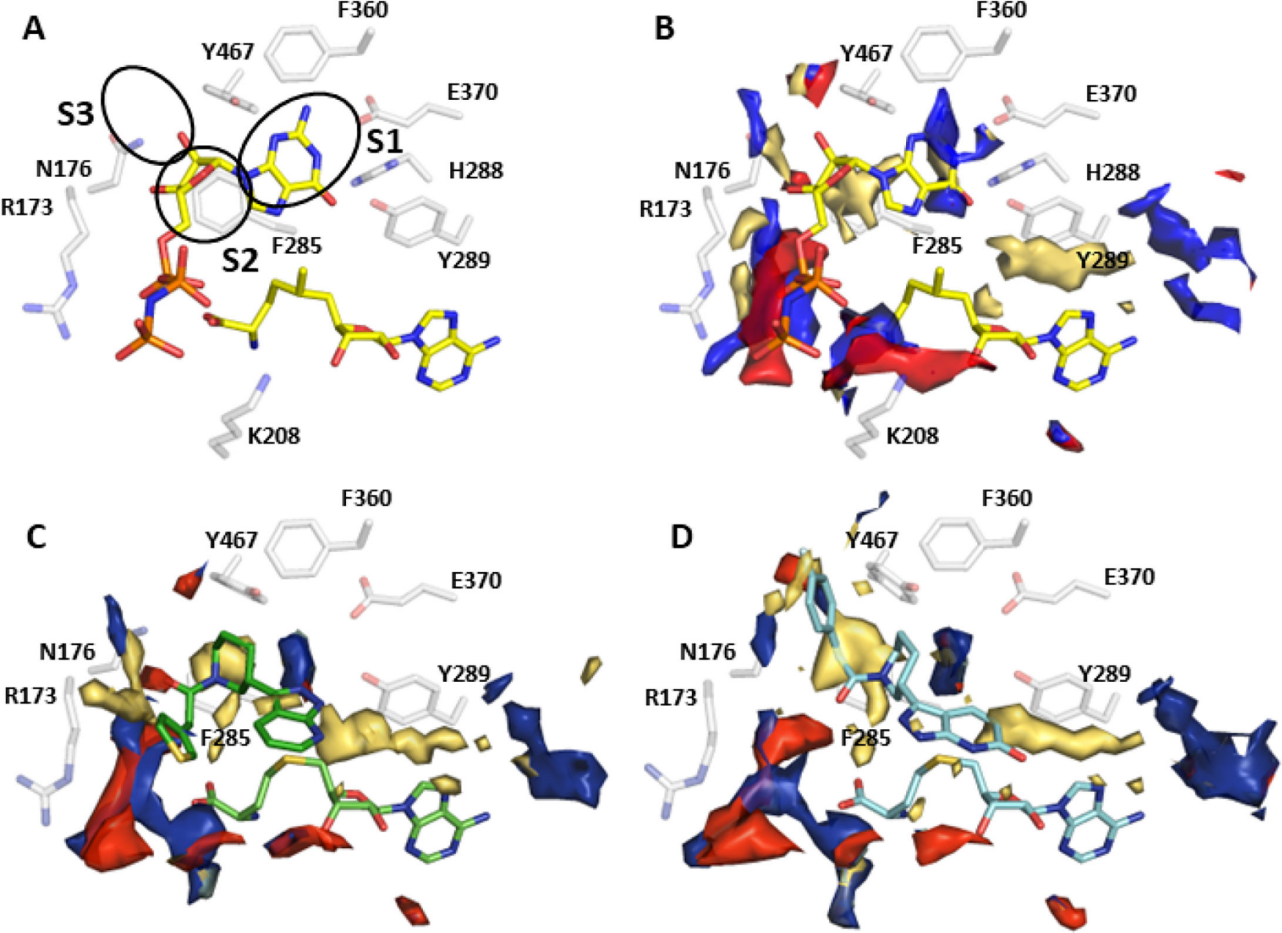
Potential compound development routes informed by MIF-based structures. The GRID MIFs calculated for the RNMT polypeptide chain (with corresponding energy cut-offs in brackets) are contoured yellow (hydrophobic: −1.5 kcal/mol), blue (hydrogen-bond donor: −5.5 kcal/mol) and red (hydrogen-bond acceptor: −5.0 kcal/mol). (**A**) Sinefungin, (**B**) cap analogue with amine modified to methyl group, (**C**) SAH-DDD1060606 and (**D**) SAH-DDD1870799 crystal structures. Abbreviations: MIF, molecular interaction field; RNMT, RNA guanine-7 methyltransferase; SAH, S-adenosylhomocysteine.

Figure 10A and B shows the calculated FLAP maps for the cap analogue (GMP-PnP) binding to RNMT. The observed polar back pocket requires a hydrogen bond donor (guanine: position 2) to interact with E370 that is bordered by hydrophobic regions (pocket S1), while the position of the 3′O (ribose) coordinates well with the requirement for a hydrogen bond acceptor to generate the hydrogen bond with N176. The DDD1060606 piperidine (Figure 10C), meanwhile, matches the hydrophobic and the hydrogen bond acceptor requirements introduced by F285 and N176, respectively (pocket S2). It is worth noting that neither the pyridine nor the thiophene can form favourable interactions with the protein, while the former sits above the thioether group of SAH. In comparison, the MIFs calculated for DDD1870799 (Figure 8D) suggest that the fluoro-phenyl moiety is responsible for the ligand binding via the interaction with a small pocket (**S3**) of high hydrophobicity. This interaction, by displacement of disorganised water molecules [41], is likely to be responsible for the significant gain in net enthalpic change ΔΔH measured for DDD1870799. Finally, since the orientations observed for the inhibitor pyridine groups do not appear to provide any advantage in comparison with guanine from the cap, the design of ligands with an improved affinity profile will have to take into consideration substituents that may be capable of stabilising the pyridine in the S1 pocket.

The biophysical characterisation of cap binding to Avi-*Hs*RNMT-RAM described earlier (cap will only bind in the presence of either SAM or Sinefungin but not SAH) suggests that the S-methyl or amine group plays a key role. As the binding poses for cap are very similar when either SAM or Sinefungin are bound, it is unlikely that hydrogen-bond formation between the amine group and the guanine base is the driver. Instead, it is likely that the common steric effects of the amine and S-methyl groups as seen in the crystal structures are important factors for stabilisation. It is only when the Sinefungin or SAM is in place that the interactions observed in the cap structure (polar with H288, Y289, E370, Y467 and Pi stacking with F285) become important. Thus, in the presence of SAH, any new inhibitor ligand should ideally carry features that consider the steric effect of the methyl on SAM to permit the guanine-mimicking group access to the S1 pocket.

## Discussion

Despite a growing interest in *Hs*RNMT-RAM as a valuable target to either inhibit the proliferation and increase the apoptosis of cancer cells [14,16,20,21] or to prevent eukaryotic virus proliferation [42], the lack of understanding of the enzymatic mechanism limits the development of selective and efficient drugs. Here, we present a comprehensive overview of the enzymatic mechanism of *Hs*RNMT monomer and in complex with its co-factor RAM. This characterisation is used as a basis to discuss *Hs*RNMT-RAM druggability through the description of two inhibitors targeting the cap pocket.

In our manuscript, we describe a label-free RapidFire mass spectrometry assay for the enzymatic characterisation of *Hs*RNMT-RAM. Although our data are robust, we observed significant differences compared with the recently published data [43]. Specifically, there were 10- and 40-fold differences in the K_m_ values for SAM and RNA, respectively. There are many potential sources for these differences. These include detail variation in the expression systems for the protein, the presence or absence of RAM and divergence in the presence or absence of a small RNA attached to the 3′ end of the cap substrate. Despite these differences, we report comparable IC_50_ values for Sinefungin, which differ by less than two-fold.

Biophysical characterisation of substrate binding shows an ordered reaction with SAM required to bind first to enable subsequent cap binding. While an ordered reaction for RNMT has been made [10], the observed order of substrate addition is reversed to the prediction.

**Figure 11:**
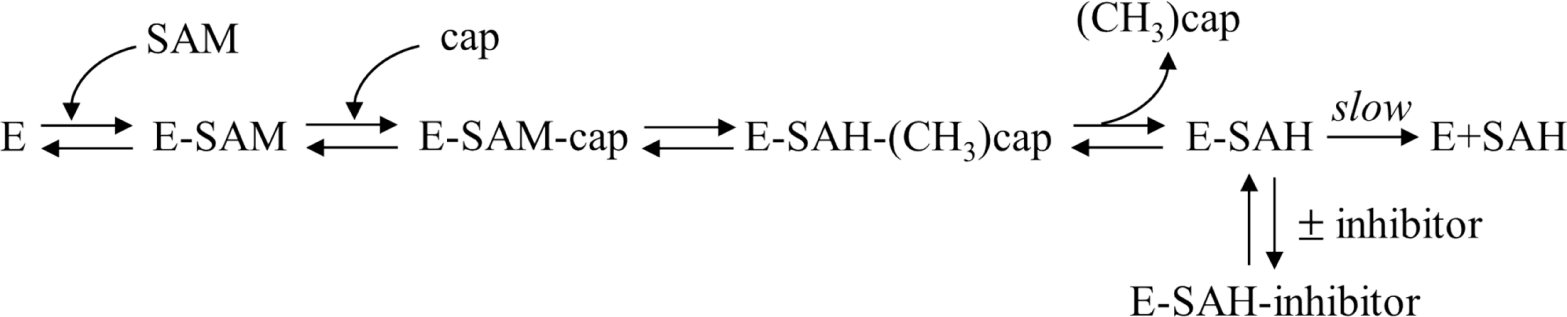
Diagram summarising the ordered bi-bi mechanism of enzymatic activity of RNMT and the enzyme-product stabilisation driven by binding of the compounds resulting in the observed uncompetitive inhibition. Abbreviation: RNMT, RNA guanine-7 methyltransferase.

We report the structure of two small molecule inhibitors of RNMT as result of a diversity-driven hit discovery campaign. These molecules behave biochemically as uncompetitive inhibitors with respect to both cap and SAM despite the structural biology data leading to an expectation of competitive inhibition with cap. The biophysical data resolve this puzzle by demonstrating that the compounds require the presence of the enzymatic product, SAH, in RNMT to bind efficiently. This stabilisation of enzyme–product complex explains the competitive binding resulting in uncompetitive inhibition. The model we propose for the behaviour of RNMT in binding its substrates and the compounds is presented (Figure 11). This is similar to the mode of action of mycophenolic acid against the inosine 5′ monophosphate dehydrogenase [44,45].

Finally, we used molecular modelling approaches to map the physicochemical properties of the cap pocket of *Hs*RNMT. This, in combination with the crystal co-complexes of the enzyme with the small molecule hits, allows us the prediction of chemical modifications that will generate better tool molecules and potentially new medicines.

## Materials and methods

Reagents were purchased from Sigma Aldrich (Gillingham, Dorset, U.K) unless otherwise stated.

### Protein production

For biochemical assays, full-length *Hs*RNMT (1-476) was cloned in the pOPC bi-cistronic plasmid as a TEV-cleavable His-MBP fusion with *Hs*RAM (1-90) and expressed in *Escherichia coli* BL21 (DE3) using LB medium. This protein is referred to as *Hs*RNMT-RAM.

For SPR, full-length *Hs*RNMT was cloned into an LIC adapted pNIC28a with an N-terminal TEV cleavable hexa-Histag and C-terminal Avitag and *Hs*RAM (1-90) into a pACYC vector and is described as Avi-*Hs*RNMT-RAM in this manuscript. Plasmids were co-transformed into *E. coli* BL21 (DE3) in the presence of a pCDFduet-BirA vector for *in vivo* biotinylation and grown in TB autoinduction media. For crystallography, the *Hs*RNMT catalytic domain (165–476, Δ416–455 GLGC) was cloned into pET15bTEV and expressed as a TEV-cleavable His tag fusion in *E. coli* BL21 DE3 using ZY autoinduction medium.

Both *Hs*RNMT full-length proteins (1-476) in complex with *Hs*RAM (1-90) (*Hs*RNMT-RAM and Avi-*Hs*RNMT-RAM) were purified using the same procedure. Harvested cells were re-suspended in a lysis buffer comprising 20 mM HEPES, 175 mM NaCl, 25 mM KCl, 10% glycerol, 0.5 mM TCEP, pH 7.5 supplemented with DNAse and EDTA-free protease inhibitors (Sigma) before lysis at 30 psi with a cell disruptor (Constant Systems). *Hs*RNMT-RAM was initially purified by Ni-NTA affinity chromatography (HisTrap FF 5 mL, Cytiva) in the same buffer and eluted with an imidazole gradient. Imidazole was then quickly removed from the fractions of interest using a HiPrep 26/10 desalting column in the same buffer. The desalted fractions were later incubated with the TEV protease (1:20) overnight at 4°C prior to a second purification by Ni affinity chromatography performed in the same conditions. The flowthrough, containing the target protein, was concentrated and purified further with gel filtration chromatography on a Superdex 75 26/60, pre-equilibrated with the same buffer. Monomeric fractions were pooled and concentrated to 15 mg/mL before snapshot freezing in liquid nitrogen. The purification of *Hs*RNMT catalytic domain (165–476) Δ416–455 GLGC was purified in a similar way with the following buffer: 20 mM HEPES, 350 mM NaCl, 50 mM KCl, 10% glycerol, 0.5 mM TCEP, pH 7.5.

### MTase-Glo screen

A selection of 48,806 diverse drug-like compounds from our internal compound library were tested for inhibition against *Hs*RMNT-RAM at a single concentration, using MTase-Glo, a bioluminescent-based assay from Promega (Madison, WI, U.S.A.). Briefly, compounds were incubated with 5 nM *Hs*RNMT-RAM, 2 µM SAM and 2 µM cap, using buffer conditions specified in the MTase-Glo assay kit, for 60 minutes. Following this incubation, the MTase-Glo Reagent and Detection solution (at ratios of 5:2 and 1:1 of the final assay volume, respectively) were added to each well and the signal allowed to develop for a further 30 minutes in the dark. The resulting luminescence was read on a PHERAstar FS (BMG). A hit was defined as percent effect was greater than mean + 3 standard deviations of the screen results (50.8%). Following this screen, compounds that had been identified as hits were confirmed by retesting at 10-point dose response in the RapidFire assay. Potency was calculated by fitting data to a four-parameter logistical fit (model 203) using Sigmaplot Version 14.0.3.192. Data are presented as the mean of three independent experiments with 95% confidence intervals (95% CI).

### RapidFire assay

An endpoint 384-well plate assay for *Hs*RMNT-RAM activity was developed using the Agilent RapidFire 365 high-throughput system with integrated SPE interfaced with the Agilent 6740 triple quadrupole mass spectrometer. The assay was performed by mixing 5 nM *Hs*RNMT-RAM in buffer (20 mM Tris, pH 8.0, 50 mM NaCl) supplemented with 1 mM EDTA, 1 mM DTT, 0.1 mg/mL BSA, 0.005% Nonidet P40 (Roche) and 3 mM MgCl2 with 2 µM of substrates (G(5′)ppp(5′)G Sodium Salt (cap) [New England Biolabs, Ipswich, MA]) and S-(5′-Adenosyl)-L-methionine chloride dihydrochloride (SAM [Cayman Chemical, Ann Arbor, MI]). The enzymatic reaction incubated for 60 minutes at room temperature followed by quenching with 80 μL 1% formic acid (VWR, Radnor, PA) containing 0.03 µg/µL S-adenosylhomocysteine-d4 (d4SAH [Cambridge Bioscience, Cambridge, U.K]). Plates were centrifuged at 500×g for 1 minute after every addition and sealed using the PlateLoc thermal microplate sealer before analysis on the RapidFire. Samples were loaded onto the RapidFire system, and analysis was performed as described for the identification of inhibitors of the SARS-CoV-2 guanine-N7-methyltransferase [32].

### K_m_ determinations

Experiments to determine the K_m_^app^ values were carried out using a two-fold dilution of cap or SAM from 40 to 0.08 µM and a saturating concentration (40 µM) of SAM (K_m_^app^ cap) or cap (K_m_^app^ SAM). *Hs*RNMT-RAM was used at 5 nM. Data were analysed to calculate the initial velocity (V0). K_m_^app^ and Vmax values were determined by nonlinear regression analysis fitted to the Michaelis–Menten equation [46] using Sigmaplot Version 14.0.3.192. Data are presented as the mean of three independent experiments with 95% CI.

### Mode of inhibition experiments

Mode of inhibition experiments were carried out by testing enzymatic activity at a range of compound and substrate concentrations. Compounds were tested at five different concentrations selected around the pIC_50_ against five substrate concentrations (between 2.2 and 20 µM). The data were fitted globally to equations describing inhibition [47] to determine the respective kinetic constants. All analyses were performed in Sigmaplot Version 14.0.3.192.

Best fit was determined using Akaike information criterion corrected [48], as well as reference to the standard error of the fit [49]. Where relevant, data are presented as the mean of three independent experiments with 95% CI.

### SPR

Studies of binding kinetics were performed on a Biacore™ 8K + instrument (GE Healthcare). Full-length *Hs*RNMT complexed to HsRAM 1–90 (Avi-*Hs*RNMT-RAM) was immobilised in HBS-P + buffer (10 mM HEPES, 150 mM NaCl, 0.5 mM TCEP, 0.05% BSA and 0.05% Tween 20) on a Series S high-affinity streptavidin (SA) sensor chip by C-terminal Avi-tagcapture, to an immobilisation level of 3300 RU. Compounds were tested in duplicate in a three-fold, eight-point serial dilution series in immobilisation buffer supplemented with 1% DMSO, with a flow rate of 30 μL min^-1^, a contact time of 60 s and a dissociation time of 120 s at 25°C.

All monitored binding resonance signals were referenced with responses from unmodified reference surfaces, corrected for bulk shifts arising from differences in DMSO concentrations between samples and running buffer (solvent correction) and blank-referenced. Analysis was performed using the Biacore™ Insight Evaluation Software 2.0 with data fitted using the Langmuir 1:1 model.

### CAP (GpppG) titration

The full-length *Hs*RNMT complexed with HsRAM 1–90 (Avi-*Hs*RNMT-RAM) was immobilised onto a Series S high-affinity SA sensor chip via C-terminal Avi-tag capture in HBS-P + buffer (10 mM HEPES, 150 mM NaCl, 0.5 mM TCEP) to an immobilisation level of 2200 RU, and the titration was performed at 15°C.

### ITC

Full-length *Hs*RNMT-RAM was dialysed overnight at 4°C against 25 mM HEPES pH 7.5, 150 mM NaCl, 1 mM TCEP supplemented with 10 μM Sinefungin or 10 μM SAH. Working stock for ligands and protein complex was prepared at the required concentration by diluting the stocks with the dialysis buffer. The DMSO concentrations in the cell and syringe solutions were matched to 2.5% for the compound (DDD1060606 or DDD1870799) titrations. Experiments were performed using 13 injections of 3 μL of ligand using Malvern PEAQ-ITC system at 25°C, at 750 rpm with reference power set to 7 μcal/s. The thermograms were analysed using MicroCal PEAQ-ITC Analysis Software, blanked to ligand control and fit to One Set of Sites Model.

### Crystallisation and structure determination

*Hs*RNMT (165-476) Δ416–455 GLGC (*Hs*RNMT) was buffer exchanged in PIPES 20 mM, NaCl 200 mM, glycerol 10% v/v, TCEP 1 mM, pH 6.5 using Zeba 10 kDa desalting columns (Thermofisher) and concentrated to 17 mg/mL. Co-complexes structures were achieved by mixing SAH (1 mM) with the appropriate inhibitor (5 mM), whereas the crystallisation of cap was only possible by a combination of Sinefungin (1 mM) with GMP-PnP (10 mM) and 2 mM MgCl_2_. *Hs*RNMT was crystallised at 17°C using the sitting drop vapor diffusion method by mixing 200 nL of the protein solution mixed with 200 nL of a reservoir solution containing 0.1M MES pH 6.3–6.9, 50 mM Na_2_SO_4_, 18–25% PEG 6000. Crystals grew in 48 hours and were flash-frozen in liquid nitrogen in the reservoir solution supplemented with 28% glycerol. Data sets were collected at 100K at beamline synchrotron ESRF-ID23 or using a Rigaku M007HF copper‐anode generator fitted with Varimax Cu‐VHF optics and a Saturn 944HG + CCD detector. Data were processed using XDS [50] in P21 and scaled using Aimless [51] to a resolution between 2.0 and 2.5 Å. The following structure solution and refinement activities were performed. The initial phases were determined by molecular replacement using Phaser [52] from the CCP4 suite [53] using the human catalytic domain *Hs*RNMT (pdb code 5E8J) with *Hs*RAM removed and the segment 416–455 as the search model. The initial density maps were further improved by solvent flattening and histogram matching using RESOLVE [54] as implemented in the Phenix suite [55]. The model was refined through iterative cycles of model building and computational refinement with COOT [56] and Phenix Refine (Phenix suite). Data measurements and model refinement statistics are presented in Supplementary Table S1. Coordinate files and associated experimental data have been deposited in the Protein Data Bank with accession codes 8Q9W, 8Q69 and 8Q8G.

## Supplementary material

online supplementary figure 1.

## Data Availability

All crystallographic data are deposited in the Protein Data Bank. All other data are contained within the manuscript.

## Abbreviations

GMP-PnP: guanosine 5′-[β, γ-imido]triphosphate
ITC: isothermal titration calorimetry
MIF: molecular interaction field
RAM: RNMT-activating miniprotein
RNMT: RNA guanine-7 methyltransferase
SAH: S-adenosylhomocysteine
SAM: S-adenosylmethionine
SPR: surface plasmon resonance
