# Correction: Characterisation of RNA guanine-7 methyltransferase (RNMT) using a small molecule approach

**DOI:** 10.1042/BCJ20240608_COR

**Published:** 2026-05-06

**Authors:** David W Gray, Lesley-Anne Pearson, Alain-Pierre Petit, Cesar Mendoza-Martinez, Fiona Bellany, De Lin, Sarah Niven, Rachel Swift, Thomas Eadsforth, Paul K Fyfe, Marilyn Paul, Vincent Postis, Xiao Hu, Victoria H Cowling

**Affiliations:** Drug Discovery Unit, School of Life Sciences, University of Dundee, Dundee, UK

**Keywords:** AKT/mTOR, apoptosis, autophagy, Glioma, Ivermectin

The authors of the original article “Characterisation of RNA guanine-7 methyltransferase (RNMT) using a small molecule approach” (DOI: 10.1042/BCJ20240608) would like to correct the Figure Legend of Figure 5. The authors state that the data have been incorrectly presented as as mM rather than μM, resulting in a 1000-fold under-representation of efficacy. They have confirmed that the logged data are correct and aligns with the graphs.

The requested correction has been assessed and approved by the Editorial Board. The authors declare that these corrections do not change the results or conclusions of their paper.

The corrected Figure Legend reads as follows:

Chemical structures of (A) DDD1060606 and (B) DDD1870799. DDD1060606 and DDD1870799 showed >80% inhibition of HsRNMT-RAM activity with determined pIC_50_s (negative logarithm of half-maximal inhibitory concentration [molar]) of 5.5 ± 0.2 (IC_50_ 3 μM; Figure 6D and 5.0 ± 0.2 (IC_50_ 10 μM; Figure 6G), respectively.

